# Supplementary material for: Primary material supply configurations and domestic recycling for cost-effective battery material production in the US
Source: Nat Commun. 2025 Dec 9;17:253. doi: 10.1038/s41467-025-66957-5 (PMC12783756; doi:10.1038/s41467-025-66957-5)
Supplement: Supplementary file 1 — Supplementary Information [file 41467_2025_66957_MOESM1_ESM.pdf]

Supplementary Information to the article:

Primary material supply configurations and domestic recycling for cost-effective battery material production in the US

Jannis Wesselkaemper[1,2*], Purabi Thakre[3], Alecia Ward[3], Andrew Z. Haddad[1*]

[1] Energy Storage and Distributed Resources Division, Lawrence Berkeley National Laboratory; Berkeley, CA 94720.

[2] Energy Analysis and Environmental Impacts Division, Lawrence Berkeley National Laboratory; Berkeley, CA 94720.

[3] Program Development Department, Lawrence Berkeley National Laboratory; Berkeley, CA 94720

*Corresponding authors. Emails: JannisWesselkaemper@lbl.gov, azhaddad@lbl.gov

Table of Contents

Supplementary Tables

Table 1. Battery characteristics and bill-of-material for NMC cathode active material production.
Table 2. Overview of key information and data all analyzed mining and refining facilities for lithium, cobalt, and nickel in this study: Country location of facility, name of facility (or project), life-of-mine (LOM) material supply capacity (in t) and calculated levelized material production costs up to levels of battery-grade materials (in USD/t).
Table 3. Applied metal and material prices for cost allocation-calculations of mines/refineries and recycling facilities in this study.
Table 4. Primary company data on mining, refining, and battery-grade manganese sulfate production facilities, including facility description, total-lifetime facility costs and manganese supply, and resulting average global LOM capacity-weighted manganese production costs.
Table 5. Primary company data on battery-grade lithium carbonate, lithium hydroxide, cobalt sulfate, and nickel sulfate production facilities, including facility description, total-lifetime facility costs and supply, and resulting levelized conversion costs.
Table 6. Regional conversion costs as applied in this study.
Table 7. Number of analyzed facilities and average lithium, cobalt, and nickel levelized production costs in regions as defined in this study.
Table 8. Aggregated material costs for NMC production for materials sourced according to the U.S. supply scenario as defined in this study.
Table 9. Aggregated material costs for NMC production for materials sourced according to the Europe supply scenario as defined in this study.
Table 10. Historic country shares of lithium supply from 2014-2025.
Table 11. Historic country shares of cobalt supply from 2014-2025.
Table 12. Historic country shares of nickel supply from 2014-2025.
Table 13. Historic country shares of manganese supply from 2014-2025.
Table 14. Global supply scenario and resulting average material production costs in global supply scenario.
Table 15. Aggregated material costs for NMC production for materials sourced according to the global supply scenario as defined in this study.

Table 16. Facility design characteristics, input/output data, total-lifetime costs, and levelized material production costs for the hydrometallurgical recycling facility as modelled in this study.
Table 17. Facility design characteristics, input/output data, total-lifetime costs, and levelized material production costs for the hydrometallurgical upcycling facility as modelled in this study.
Table 18. Consumption data and costs of the hydrometallurgical recycling plant.
Table 19. Consumption data and costs of the hydrometallurgical upcycling plant.
Table 20. Applied metal contents in various NMC black mass inputs based on Benchmark Mineral Intelligence data and molar mass-based recalculations.
Table 21. Calculated black mass prices (in USD/t) for various NMC ratios and metal price scenarios (baseline, moderate, minimum).
Table 22. Calculated aggregated material cost for NMC CAM production (in USD/GWh CAM) supplied by hydrometallurgical recycling and upcycling facilities for baseline, moderate, and minimum metal price scenarios.
Table 23. Total end-of-life NMC battery supply in the United States for 2025 to 2050 in a low, basic, and high supply scenario as defined in this study.
Supplementary Notes
Note 1. NMC cathode active material (CAM) production data for material cost aggregation.
Note 2. Primary company data: Global lithium, cobalt and nickel mining and refining facilities.
Note 3. By-product cost allocation methodology: Market value-based cost allocation (including recycling).
Note 4. Cost data for other CAM production input materials: Manganese, sodium hydroxide, and ammonium hydroxide.
Note 5. Conversion to battery-grade materials: Primary company data, complementary techno-economic modelling, and average regional conversion costs.
Note 6. Average regional levelized production costs and aggregated costs for U.S. and Europe supply scenarios.
Note 7. Definition of average global supply scenario costs: Global battery material supply data.
Note 8. Recycling costs: Primary company data and techno-economic modelling.
Note 9. Black mass price modelling.
Note 10. Learning effects or economies-of-scale: Wright's Law.
Note 11. U.S. end-of-life (EoL) battery supply modelling.
Supplementary References

Supplementary Table 1. Battery characteristics and bill-of-material for NMC cathode active material production. NMC CAM Data sourced from GREET.[1,2]

	CAM production data		Battery CAM chemistry					Notes
	Characteristics	Unit	NMC 95	NMC 811	NMC 622	NMC 532	NMC 111	
Battery paramete	EV type/size	miles	400	400	400	400	400	*Input data obtained from GREET*
	Energy Density	kWh/t pack	211.3	203.3	192.3	190.9	182.3	
	CAM mass share	wt% in pack	24.88	25.90	27.43	27.78	28.88	
	Energy density	kWh/t CAM	849.3	784.9	701.1	687.2	631.2	
	Active material	t CAM/GWh	1,177.5	1,274.0	1,426.4	1,455.2	1,584.2	
Material requirements	Li_2CO_3	t/t NMC CAM	0.380	0.381	0.381	0.382	0.383	*Input data obtained from GREET*
	pCAM	t/t NMC CAM	0.950	0.949	0.949	0.949	0.949	*Input data obtained from GREET*
	$CoSO_4$	t/t NMC CAM	0.040	0.159	0.320	0.332	0.522	*Recalculation based on pCAM intensity in bill-of-materials (obtained from GREET)*
	$NiSO_4$	t/t NMC CAM	1.508	1.272	0.958	0.824	0.535	
	$MnSO_4$	t/t NMC CAM	0.039	0.155	0.311	0.452	0.522	
	$CoSO_4$ x $7H_2O$	t/t NMC CAM	0.072	0.289	0.580	0.603	0.947	*Recalculation based on molar masses*
	$NiSO_4$ x $6H_2O$	t/t NMC CAM	2.561	2.160	1.626	1.400	0.909	
	$MnSO_4$ x H_2O	t/t NMC CAM	0.044	0.173	0.348	0.506	0.584	
	Co	t/t NMC CAM	0.015	0.061	0.122	0.126	0.198	*Recalculation based on molar masses*
	Ni	t/t NMC CAM	0.572	0.482	0.363	0.313	0.203	
	Mn	t/t NMC CAM	0.014	0.056	0.113	0.164	0.190	
	NaOH	t/t NMC CAM	0.846	0.845	0.845	0.845	0.845	*Recalculation based on pCAM intensity in bill-of-materials (obtained from GREET)*
	NH_4OH	t/t NMC CAM	0.118	0.118	0.118	0.118	0.118	

Supplementary Table 2. Overview of key information and data all analyzed mining and refining facilities for lithium, cobalt, and nickel in this study: Country location of facility, name of facility (or project), life-of-mine (LOM) material supply capacity (in t), and calculated levelized material production costs up to levels of battery-grade materials (in USD/t).

Nr.	Material	Country (facility)	Facility/project name	LOM supply capacity (in t)	Levelized production costs up to battery-grade material (Li_2CO_3/LiOH, $CoSO_4$, $NiSO_4$) (in USD/t)
1)	Li	Australia	Greenbushes Mine	5,178.669	4,676.2
2)	Li	Australia	Mt. Marion Lithium Project	176,000	4,941.6
3)	Li	Australia	Mt. Cattlin Lithium Project	733.947	3,789.9
4)	Li	Australia	Pilgangoora Operations	1,677.288	4,445.8
5)	Li	Australia	Mt. Holland Lithium Project	2,213.200	6,240.9
6)	Li	Australia	Finness Lithium Project	65.426	5,967.0
7)	Li	Australia	Kathleen Valley Project	2,158.335	4,813.2
8)	Li	Australia	Bald Hill Lithium-Tantalum Project	82.796	5,260.9
9)	Li	Argentina	Cauchari Lithium Brine Project	740,072	6,126.4
10)	Li	Argentina	Olaroz Lithium Project	1,310,670	5,360.6
11)	Li	Argentina	Salar del Hombre Muerto	3,599,000	6,045.6
12)	Li	Argentina	Sal de Vida Lithium Brine Project	582,719	5,908.5
13)	Li	Argentina	Cauchari-Olaroz Salars	1,452,000	7,978.5
14)	Li	Argentina	Rincon Lithium Project	165,000	5,949.1
15)	Li	Argentina	Mariana Lithium Brine Project	250,000	5,640.3
16)	Li	Brazil	Bandeira Lithium Project	339.058	7,647.4
17)	Li	Brazil	Grota do Cirilo Lithium Project	1,657.510	6,582.5
18)	Li	Chile	Salar de Atacama/La Negra	1,672,059	4,218.8
19)	Li	Chile	Salar de Atacama/Salar del Carmen	1,396,800	9,389.5
20)	Li	Canada	Georgia Lake Project	118.956	12,649.2
21)	Li	Canada	Clearwater Lithium Porject	1,137.400	11,963.3
22)	Li	Canada	North American Lithium Project	539.521	9,366.0
23)	Li	Canada	Moblan Lithium Project	867.753	9,472.5
24)	Li	Canada	PAK Lithium Project	553.731	13,690.5
25)	Li	Canada	Whabouchi Mine	1,034.382	12,853.1
26)	Li	Canada	Boardwalk Property	589.513	9,322.1
27)	Li	Canada	Kindersley Lithium Project	193.600	6,171.4
28)	Li	Canada	Rose Lithium Project	480.838	11,467.9
29)	Li	Canada	James Bay Lithium Project	834.529	8,475.3
30)	Li	Mexico	Sonora Lithium Project	600,900	5,641.7

31)	Li	USA	Thacker Pass Project	2,671,320	9,297.3
32)	Li	USA	TLC Lithium Project	1,407,080	9,639.6
33)	Li	USA	Rhyolite Ridge project	570,726	7,576.7
34)	Li	USA	Tonopah Flats Lithium Project	1,419,048	7,235.7
35)	Li	USA	Bonnie Claire Lithium Project	1,292,000	6,410.9
36)	Li	USA	Angel Island Project	1,393,400	10,875.2
37)	Li	USA	Silver Peak Operations	150,400	10,206.5
38)	Li	USA	South-West Arkansas (SWA) Project	528,000	8,314.5
39)	Li	USA	Clayton Valley Lithium Project	194,040	5,650.8
40)	Li	USA	Carolina Lithium Project	341,440	6,737.1
41)	Li	DR Congo	Manono Lithium Tailings Project	91.538	8,479.9
42)	Li	DR Congo	Manono Lithium and Tin Project	1,684.732	5,343.4
43)	Li	Ghana	Ewoyaa Lithium Project	509.695	5,780.4
44)	Li	Mali	Goulamina Lithium Project	2,314.728	4,741.6
45)	Li	Mali	Bougouni Lithium Project	287.857	6,003.0
46)	Li	Namibia	Karibib Lithium project	62,335	15,710.7
47)	Li	Namibia	Swanson Lithium Project	51.455	3,939.0
48)	Li	Zimbabwe	Arcadia Lithium Project	436.237	6,483.4
49)	Li	Zimbabwe	Zulu Lithium Project	186.959	7,303.6
50)	Li	Portugal	Barroso Lithium Project	362.800	9,456.4
51)	Li	Czech	Cinovec Project	734,650	6,992.8
52)	Li	Austria	Wolfsberg Lithium project	128,976	23,529.3
53)	Li	Finland	Keliber Lithium Project	193.853	12,331.5
54)	Li	Spain	San Jose Lithium project	395.842	7,645.3
55)	Li	Germany	Upper Rhine Valley	363.651	10,074.7
56)	Li	Germany	Zinnwald Lithium Project	597.744	8,814.8
57)	Co	DR Congo	Tenke Fungurume Mine	392,429	17,310.8
58)	Co	DR Congo	Kamoto Copper Operations	455,900	13,716.4
59)	Co	DR Congo	Boss Mining Project	186,000	10,981.1
60)	Co	USA	Idaho Cobalt Operations	9,330	36,640.7
61)	Co/Ni	USA	Eagle Mine	1,165 (Co)<br>45,343 (Ni)	26,304.1 (Co)<br>11,387.3 (Ni)
62)	Co/Ni	Canada	Sudbury Property	6,200 (Co)<br>560,000 (Ni)	40,459.5 (Co)<br>18,465.0 (Ni)
63)	Co/Ni	Canada	Baptiste Nickel Project	19,380 (Co)<br>1,619,316 (Ni)	35,864.4 (Co)<br>14,174.1 (Ni)
64)	Co/Ni	Canada	Crawford Nickel Sulphide Project	24,494 (Co)<br>1,559,904 (Ni)	24,646.6 (Co)<br>10,558.6 (Ni)
65)	Co/Ni	Canada	Shakespeare Project	1,376 (Co)<br>29,823 (Ni)	29,454.7 (Co)<br>12,962.6 (Ni)
66)	Co/Ni	Canada	Crean Hill Project	3,084 (Co)<br>88,677 (Ni)	28,789.9 (Co)<br>12,630.2 (Ni)
67)	Co/Ni	Canada	Turnagain Nickel Project	58,000 (Co)<br>982,500 (Ni)	23,441.9 (Co)<br>9,956.2 (Ni)
68)	Co/Ni	Canada	Lockerby East Property	434 (Co)<br>23,439 (Ni)	29,647.9 (Co)<br>13,059.2 (Ni)
69)	Co/Ni	Cuba	Moa Nickel Project	84,679 (Co)<br>723,552 (Ni)	32,818.6 (Co)<br>15,037.1 (Ni)
70)	Co/Ni	Papua N.G.	Ramu Nickel Cobalt Project	47,500 (Co)<br>427,900 (Ni)	16,314.2 (Co)<br>6,784.9 (Ni)
71)	Co/Ni	Australia	Sunrise Nickel Cobalt Project	86,250 (Co)<br>463,000 (Ni)	20,776.9 (Co)<br>10,388.4 (Ni)
72)	Co/Ni	Australia	Ravensthorpe	17,930 (Co)<br>538,442 (Ni)	30,606.2 (Co)<br>13,094.9 (Ni)
73)	Co/Ni	Australia	NiWest Nickel Cobalt Project	47,000 (Co)<br>627,300 (Ni)	26,168.0 (Co)<br>13,084.0 (Ni)
74)	Co/Ni	Madagascar	Ambatovy Nickel Project	98,899 (Co)<br>1,130,231 (Ni)	27,250.4 (Co)<br>12,253.0 (Ni)
75)	Ni	Indonesia	Sorowako Mine	1,798,721	12,065.2
76)	Ni	Canada	Dumont Nickel Project	1,089,529	10,879.8
77)	Ni	Canada	Bucko Lake Nickel Project	45,813	21,710.7
78)	Ni	Spain	Aguablance Nickel Copper Mineral Deposit	19,602	13,228.7
79)	Ni	Cote d'Ivoire	Samapleu and Grata Deposits Project	92,285	16,063.8

Supplementary Table 3. Applied metal and material prices for cost allocation-calculations of mines/refineries and recycling facilities in this study.

Facility	Material	Price (USD/t)	Source
Mining/refining	Co	30,000	https://tradingeconomics.com/commodity/cobalt
	Ni	15,000	https://tradingeconomics.com/commodity/nickel
	Mn	2,000	https://tradingeconomics.com/commodity/manganese
	Cu	8,000	https://tradingeconomics.com/commodity/copper
	Pt	30,000,000	https://tradingeconomics.com/commodity/platinum
	Pd	30,000,000	https://tradingeconomics.com/commodity/palladium
	Au	100,000,000	https://tradingeconomics.com/commodity/gold
	Cr	8,000	https://www.metal.com/en/markets/16
	Fe	100	https://tradingeconomics.com/commodity/iron-ore
Recycling	Li_2CO_3	13,000	https://tradingeconomics.com/commodity/lithium
	pCAM NMC 111	23,314	Metal-based material production costs (including margin 50%)
	pCAM NMC 532	21,816	Metal-based material production costs (including margin 50%)
	pCAM NMC 622	22,875	Metal-based material production costs (including margin 50%)
	pCAM NMC 811	22,545	Metal-based material production costs (including margin 50%)
	pCAM NMC 95	22,303	Metal-based material production costs (including margin 50%)
	Na_2SO_4	200	https://businessanalytiq.com/procurementanalytics/index/sodium-sulfate-price-index/

Supplementary Table 4. Primary company data on mining, refining, and battery-grade manganese sulfate production facilities, including facility description, total-lifetime facility costs and manganese supply, and resulting average global LOM capacity-weighted manganese production costs applied in this study. HPMSM = high-purity manganese sulfate monohydrate; HPEMM = high-purity electrolytic manganese metal

		Manganese production facilities		
Description	Facility/project name	Chvaletice Manganese Project	K.Hill Battery-Grade Manganese Project	High Purity Manganese Sulphate Project (supplied by Tshipi mine
	Ownership	100% Euro Manganese Inc. (Canada)	100% Giyani Metals Corp. (Canada)	Refinery: 100% Jupiter Mines Ltd. (Australia)<br>Mine: 49.9% Jupiter Mines Ltd (Australia), 50.1% Ntsimbintle Mining (South Africa)
	Location	Czech Republic	Botswana	Refinery: (North America)<br>Mine: South Africa
	Source/company report	*'Technical Report and Feasibility Study for the Chvaletice Manganese Project, Czech Republic'* (September 9, 2022) *	*'K.Hill Battery-Grade Manganese Project Feasibility Study - National Instrument 43-101 Technical Report'* (November 14, 2022)	*'ASX Announcement: Scoping Study – High Purity Manganese Sulphate Project'* (March 13, 2024)
	Manganese source/process input	Manganese-bearing waste/tailings from a decommissioned mine	Manganese oxide/carbonate ores	30% Mn ore supplied from Tshipi mine (Mn ore concentrated from run-of-mine ore at Tshipi mine before shipping)
	Process description (high-level)	Magnetic separation, sulfuric acid leaching, purification/electrowinning, Acid dissolution of HPEMM, HPMSM crystallization	Ore crushing/grinding, SO_2/ sulfuric acid leaching, purification/base metal precipitation, fluoride polishing, HPMSM crystallization	Ore grinding/milling, acid leaching, purification by removal of iron, calcium, magnesium etc., HPMSM crystallization
	Product	HPMSM; HPEMM	HPMSM	HPMSM
	LOM (years)	26	14	25
	LOM supply capacity (t Mn)	1,171,911	262,859	682,581
Costs	CapEx (million USD)	757.4	283.7	430.1
	OpEx (million USD)	5,595.7	1,369.2	2,101.5
	SusEx (million USD)	117.0	18.5	45.0
	Closure costs (million USD)	2.0	5.1	-
	Levelized Mn production costs (USD/t Mn in $MnSO_4$)	1,881.0	2,073.1	1,227.0
Average global LOM capacity-weighted Mn production costs		1,694.02 USD/t Mn		

Supplementary Table 5. Primary company data on battery-grade lithium carbonate, lithium hydroxide, cobalt sulfate, and nickel sulfate production facilities, including facility description, total-lifetime facility costs and supply, and resulting levelized conversion costs.

		Battery-grade material production (conversion) facilities			
Description	Facility/project name	Bécancour Lithium Refinery	Thunder Bay Lithium Processing Facility (Lake Superior Lithium Project)	Ontaria Cobalt Refinery Project	Awaruite Refinery
	Ownership	Lithium Universe Ltd. (Canada)	Avalon Advanced Materials Inc. (Canada)	Electra Battery Materials Corp. (Canada)	FPX Nickel Corp. (Canada)
	Location	Canada	Canada	Canada	Canada
	Source/company report	*'The Bécancour Lithium Refinery Definitive Feasibility Study'* (February 2025)	*'Preliminary Economic Assessment ("PEA") for its Lithium Hydroxide Processing Facility project in Thunder Bay, Ontario, Canada'* (September 3, 2024)	*'First Cobalt Refinery Project - Association for the Advancement of Cost Engineering (AACE) Class 3 Feasibility Study* (July 9, 2020)	*'AWARUITE REFINERY SCOPING STUDY REPORT'* (March 25, 2025)
	Process input material	Spodumene concentrate (5.5-6% Li_2O)	Spodumene concentrate (5.5-6% Li_2O)	Cobalt hydroxide $Co(OH)_2$	Awaruite, MHP
	Process description (high-level)	Calcination to beta spodumene, sulfuric acid leaching, purification by precipitation/ion exchange, Li_2CO_3 precipitation with soda ash, crystallization	Calcination to beta spodumene, slurry repulping with soda ash, carbonate pressure leaching, conversion with lime, ion exchange, crystallization of LiOH	Sulfuric acid leaching, impurity removal by precipitation and solvent extraction, cobalt solvent extraction, sulfuric acid stripping, $CoSO_4$ crystallization	Sulfuric acid leaching (atmospheric/pressure), purification (Cu/Co), nickel solvent extraction, $NiSO_4$ crystallization
	Product	Li_2CO_3	LiOH monohydrate	$CoSO_4$	$NiSO_4$ (+ by-products)
	Facility lifetime	20 years	30 years	11 years	40 years
	Lifetime supply capacity	365,400 t LCE	792,000 t LCE	56,053 t Co	1,280,000 t Ni
Costs	CapEx (million USD)	549.0	910.5	56.0	424.0
	OpEx (million USD)	1,436,4	3,508.2	359.1	2,045.5
	SusEx (million USD)	100.0	46.5	0.6	40.0
	Closure costs (million USD)	-	30.0	5.6	42.0
	Levelized production costs	5,707.1 USD/t LCE	5,675.8 USD/t LCE	7,516.2 USD/t Co	1,993.4 USD/t Ni

Supplementary Table 6. Regional conversion costs as applied in this study. *Average of levelized conversion costs of both Canadian spodumene concentrate conversion facilities in the data set of primary company conversion data in this study.

Material	Region (as defined in this study)	Applied regional levelized conversion costs (USD/t)	Regional conversion costs assumptions
Li	Australia	2,456.0	Low
	Africa	2,456.0	Low
	Canada	5,691.4*	High
	Europe	5,691.4*	High
	Mexico	-	*(no conversion required)*
	South America	2,456.0	Low
	USA	-	*(no conversion required)*
Co	Australia	5,844.2	Low
	Canada	7,516.2	High
	Cuba	5,844.2	Low
	DR Congo	5,844.2	Low
	Madagascar	5,844.2	Low
	Papua New Guinea	5,844.2	Low
	USA	7,516.2	High
Ni	Australia	1,549.9	Low
	Africa	1,549.9	Low
	Canada	1,993.4	High
	Cuba	1,549.9	Low
	Indonesia	1,549.9	Low
	Papua New Guinea	1,549.9	Low
	Spain/Europe	1,993.4	High
	USA	1,993.4	High

Supplementary Table 7. Number of analyzed facilities and average lithium, cobalt, and nickel levelized production costs in regions as defined in this study. By-product cost shares are determined based on market value-based cost allocation (see also Supplementary Note 3). All cost data entail levelized material production costs up to final products for battery cathode active material (CAM) production (Li_2CO_3/LiOH monohydrate; $CoSO_4$ heptahydrate; $NiSO_4$ hexahydrate). *By-products which were considered in the market value-based cost allocation methodology (see Supplementary Note 3).

Material	Region (as defined in this study)	Number of facilities analyzed	Average levelized production costs (weighted by LOM capacity) (USD/t)	By-products*
Li	Australia	9	4,632.3	-
	Africa	9	5,384.4	-
	Canada	10	10,582.5	-
	Europe	7	9,418.0	-
	Mexico	`1	5,641.7	-
	South America	11	6,584.3	-
	USA	10	8,603.0	-
Co	Australia	3	23,618.7	Ni
	Canada	7	27,011.3	Cu, Ni, Au, Pt, Pd, Fe, Cr
	Cuba	1	32,818.6	Fe, Ni
	DR Congo	3	14,588.3	Cu
	Madagascar	1	27,250.4	Ni
	Papua New Guinea	1	16,314.2	Ni
	USA	2	35,493.4	Ni, Cu, Au
Ni	Australia	3	12,321.35	Co
	Africa	2	12,540.70	Cu, Co
	Canada	9	12,261.59	Cu, Co, Au, Pt, Pd, Fe, Cr
	Cuba	1	15,037.14	Fe, Co
	Indonesia	1	12,065.21	-
	Papua New Guinea	1	6,784.91	Co
	Spain/Europe	1	13,228.74	Cu
	USA	1	11,387.34	Cu, Co

Supplementary Table 8. Aggregated material costs for NMC production for materials sourced according to the U.S. supply scenario as defined in this study.

U.S. supply scenario					
**CAM chemistry**	**NMC 95**	**NMC 811**	**NMC 622**	**NMC 532**	**NMC 111**
Aggregated material costs (USD/GWh CAM)	12,688,324	14,586,272	17,621,617	17,521,590	21,236,216

Supplementary Table 9. Aggregated material costs for NMC production for materials sourced according to the Europe supply scenario as defined in this study.

Europe supply scenario					
**CAM chemistry**	**NMC 95**	**NMC 811**	**NMC 622**	**NMC 532**	**NMC 111**
Aggregated material costs (USD/GWh CAM)	14,141,212	15,458,176	17,547,101	17,252,808	19,656,024

Supplementary Table 10. Historic country shares of lithium supply from 2014-2025. Note: Global lithium resources are estimated to amount to 115 Mt in 2024. Data sourced from United States Geological Survey's (USGS) Mineral Commodity Summaries reports from 2015 to 2025.[3]

Country	Global annual lithium supply shares											Country reserves (in t)
	2024	2023	2022	2021	2020	2019	2018	2017	2016	2015	2014	by 2024
Australia	36.7%	45.0%	51.2%	51.7%	48.1%	52.3%	61.9%	58.0%	36.8%	44.8%	42.0%	7,000,000
Chile	20.4%	20.3%	26.0%	26.4%	26.1%	22.4%	17.9%	20.6%	37.6%	33.3%	36.3%	9,300,000
China	17.1%	17.5%	15.5%	13.1%	16.1%	12.6%	7.5%	9.9%	6.1%	6.3%	7.3%	3,000,000
Argentina	7.5%	4.2%	4.5%	5.6%	7.2%	7.3%	6.7%	8.3%	15.3%	11.4%	10.1%	4,000,000
Brazil	4.2%	2.6%	1.8%	1.6%	1.7%	2.8%	0.3%	0.3%	0.5%	0.6%	0.5%	390,000
Canada	1.8%	1.6%	0.4%	0.0%	0.0%	0.2%	2.5%	0.0%	0.0%	0.0%	0.0%	1,200,000
Zimbabwe	9.2%	7.3%	0.7%	0.7%	0.5%	1.4%	1.7%	1.2%	2.6%	2.9%	2.8%	480,000
Portugal	0.2%	0.2%	0.3%	0.8%	0.4%	1.0%	0.8%	1.2%	1.1%	0.6%	0.9%	60,000
Other	3.1%	1.4%	0.0%	0.1%	0.0%	0.0%	0.6%	0.7%	0.0%	0.0%	0.1%	4,570,000
**Total**	**100%**	**100%**	**100%**	**100%**	**100%**	**100%**	**100%**	**100%**	**100%**	**100%**	**100%**	**30,000,000**
**Global supply (t/a)**	**240,000**	**204,000**	**146,000**	**107,000**	**82,500**	**86,000**	**95,000**	**69,000**	**38,000**	**31,500**	**31,700**	**-**

Supplementary Table 11. Historic country shares of cobalt supply from 2014-2025. Note: Global cobalt resources are estimated to amount to 25 Mt by 2024. Data sourced from United States Geological Survey's (USGS) Mineral Commodity Summaries reports from 2015 to 2025.[3] (*included in 'Other')

Country	Global annual cobalt supply shares											Country reserves (in t)
	2024	2023	2022	2021	2020	2019	2018	2017	2016	2015	2014	by 2024
DR Congo	75.9%	73.5%	73.1%	72.1%	69.0%	69.4%	70.3%	60.8%	58.2%	50.0%	51.2%	6,000,000
Indonesia	9.7%	8.0%	4.9%	1.6%	0.8%	*	*	*	*	*	*	640,000
Russia	3.0%	3.7%	4.7%	4.8%	6.3%	4.4%	4.1%	4.9%	5.0%	4.9%	5.1%	250,000
Canada	1.6%	1.8%	1.6%	2.6%	2.6%	2.3%	2.4%	3.2%	3.9%	5.5%	5.3%	220,000
Philippines	1.3%	1.6%	2.0%	2.2%	3.2%	3.5%	3.1%	3.8%	3.7%	3.4%	3.7%	260,000
Australia	1.2%	2.2%	2.9%	3.2%	4.0%	4.0%	3.3%	4.2%	5.0%	4.8%	4.9%	1,700,000
Cuba	1.2%	1.4%	1.9%	2.4%	2.7%	2.6%	2.4%	4.2%	3.8%	3.4%	3.0%	500,000
Papua New Gui.	1.0%	0.9%	1.5%	1.8%	2.1%	2.0%	2.2%	2.8%	2.0%	*	*	62,000
USA	0.1%	0.2%	0.3%	0.4%	0.4%	0.3%	0.3%	0.5%	0.6%	0.6%	0.1%	70,000
Other	5.2%	7.0%	7.5%	9.1%	9.4%	11.7%	12.2%	16.1%	18.4%	28.0%	26.7%	1,298,000
**Total**	**100%**	**100%**	**100%**	**100%**	**100%**	**100%**	**100%**	**100%**	**100%**	**100%**	**100%**	**11,000,000**
**Global supply (t/a)**	**290,000**	**238,000**	**197,000**	**165,000**	**142,000**	**144,000**	**148,000**	**120,000**	**110,000**	**126,000**	**123,000**	**-**

Supplementary Table 12. Historic country shares of nickel supply from 2014-2025. Note: Global nickel resources are estimated to amount to 350 Mt by 2024. Data sourced from United States Geological Survey's (USGS) Mineral Commodity Summaries reports from 2015 to 2025.[3]

Country	Global annual nickel supply shares											Country reserves (in t)
	2024	2023	2022	2021	2020	2019	2018	2017	2016	2015	2014	by 2024
Indonesia	59.5%	54.1%	48.3%	38.1%	30.7%	32.7%	25.3%	16.0%	9.5%	5.7%	7.2%	55,000,000
Philippines	8.9%	11.0%	10.6%	14.2%	13.3%	12.4%	14.4%	16.9%	16.6%	24.3%	21.3%	4,800,000
New Caledonia	3.0%	6.2%	6.1%	6.8%	8.0%	8.0%	9.0%	10.0%	9.9%	8.2%	7.3%	7,100,000
Russia	5.7%	5.6%	6.7%	7.5%	11.3%	10.7%	11.3%	9.9%	10.6%	11.8%	9.8%	8,300,000
Canada	5.1%	4.2%	4.4%	4.9%	6.7%	6.9%	7.3%	9.9%	11.3%	10.3%	9.6%	2,200,000
China	3.2%	3.1%	3.5%	4.0%	4.8%	4.6%	4.6%	4.8%	4.7%	4.1%	4.1%	4,400,000
Australia	3.0%	4.0%	4.7%	5.5%	6.7%	6.1%	7.1%	8.3%	9.8%	9.7%	10.0%	24,000,000
Brazil	2.1%	2.2%	2.7%	2.8%	3.1%	2.3%	3.1%	3.6%	7.7%	7.0%	4.2%	16,000,000
US	0.2%	0.4%	0.5%	0.7%	0.7%	0.5%	0.7%	1.0%	1.2%	1.2%	0.2%	310,000
Other	9.3%	9.1%	12.5%	15.5%	14.8%	15.8%	17.2%	19.6%	18.8%	17.7%	26.4%	7,890,000
**Total**	**100%**	**100%**	**100%**	**100%**	**100%**	**100%**	**100%**	**100%**	**100%**	**100%**	**100%**	**130,000,000**
**Global supply (t/a)**	**3,700,000**	**3,750,000**	**3,270,000**	**2,730,000**	**2,510,000**	**2,610,000**	**2,400,000**	**2,160,000**	**2,090,000**	**2,280,000**	**2,450,000**	**-**

Supplementary Table 13: Historic country shares of manganese supply from 2014-2025. Data sourced from United States Geological Survey's (USGS) Mineral Commodity Summaries reports from 2015 to 2025.[3]

Country	Global annual manganese supply shares											Country reserves (in t)
	2024	2023	2022	2021	2020	2019	2018	2017	2016	2015	2014	by 2024
S. Africa	37.0%	37.2%	36.9%	35.8%	34.4%	29.6%	30.7%	31.2%	33.8%	33.7%	29.2%	560,000,000
Gabon	23.0%	22.9%	23.6%	21.6%	17.5%	12.8%	12.3%	12.7%	10.3%	11.5%	10.4%	61,000,000
Australia	14.0%	14.6%	15.4%	16.2%	17.6%	16.2%	18.4%	16.3%	14.3%	14.0%	17.1%	500,000,000
Ghana	4.1%	4.2%	4.3%	4.7%	3.4%	7.9%	7.2%	4.7%	3.5%	2.4%	2.3%	13,000,000
China	3.9%	3.9%	3.8%	4.9%	7.1%	6.8%	6.3%	9.8%	14.8%	17.1%	16.9%	280,000,000
India	4.0%	3.8%	3.6%	2.3%	3.3%	4.1%	5.1%	4.2%	4.7%	5.1%	5.3%	34,000,000
Brazil	3.0%	3.0%	3.2%	2.7%	2.6%	8.9%	6.9%	6.7%	6.9%	6.2%	5.8%	270,000,000
Malaysia	2.1%	2.1%	1.2%	1.8%	1.8%	2.0%	2.1%	2.8%	1.7%	1.1%	2.1%	-
Other	9.1%	8.3%	8.1%	10.0%	12.2%	11.7%	10.9%	11.6%	10.0%	8.7%	10.7%	-
**Total**	**100%**	**100%**	**100%**	**100%**	**100%**	**100%**	**100%**	**100%**	**100%**	**100%**	**100%**	**1,718,000,000**
**Global supply (t/a)**	**20,000,000**	**19,600,000**	**19,800,000**	**20,100,000**	**18,900,000**	**19,600,000**	**18,900,000**	**17,300,000**	**15,700,000**	**17,500,000**	**17,800,000**	**-**

Supplementary Table 14. Global supply scenario and resulting average material production costs in global supply scenario. *Average material production costs are calculated by LOM capacity-weighted regional average production costs.

Average global supply scenario		
**Material**	**Country supply shares (%)**	**Average material costs* (USD/t)**
LCE	36.7% Australia, 20.4% Chile, 9.2% Zimbabwe, 7.5% Argentina, 4.2% Brazil, 1.8% Canada, 0.2% Portugal, 20.1% other	5,853.4
Co	75.9% DR Congo, 1.6% Canada, 1.2% Australia, 1.2% Cuba, 20.1% other	17,107.3
Ni	59.5% Indonesia, 5.1% Canada, 3.0% Australia, 32.4% other	12,144.6
Mn	100% global (average global production costs)	1,694.0
NaOH	100% global (market price)	345.0
NH_4OH	100% global (market price)	1,200.0

Supplementary Table 15. Aggregated material costs for NMC production for materials sourced according to the global supply scenario as defined in this study.

Average global supply scenario					
**CAM chemistry**	**NMC 95**	**NMC 811**	**NMC 622**	**NMC 532**	**NMC 111**
Aggregated material costs (USD/GWh CAM)	11,639,451	12,296,971	13,330,354	12,957,454	14,030,689

Supplementary Table 16. Facility design characteristics, input/output data, total-lifetime costs, and levelized material production costs for the hydrometallurgical recycling facility as modelled in this study. *Levelized material production costs are based on market value-based cost allocation.

Hydrometallurgical Recycling Facility						
Facility outlet/ process design	Output metal ratio	NMC 95	NMC 811	NMC 622	NMC 532	NMC 111
	Lifetime	20 years				
	Availability	8000 hr/year				
	Capacity	35,000 t black mass/year				
	Input black mass ratio	NMC 95	NMC 811	NMC 622	NMC 532	NMC 111
Facility lifetime output (t)	Li_2CO_3	165,000.0	165,000.0	165,000.0	165,000.0	165,000.0
	$CoSO_4$	19,127.8	84,157.9	153,022.4	153,022.4	255,037.3
	$NiSO_4$	725,758.8	595,894.8	458,374.0	381,978.3	254,652.2
	$MnSO_4$	10,729.5	47,207.5	85,836.3	128,754.4	143,060.4
	Li	30,998.8	30,998.8	30,998.8	30,998.8	30,998.8
	Co	7,273.1	32,000.0	58,184.8	58,184.8	96,974.7
	Ni	275,252.4	226,000.0	173,843.6	144,869.7	96,579.8
	Mn	6,780.1	29,830.8	54,240.5	81,360.8	90,400.9
Facility lifetime costs (million USD)	CapEx/SusEx	1,347.6	1,347.6	1,347.6	1,347.6	1,347.6
	OpEx	5,625.5	5,627.4	5,587.1	5,323.7	5,633.3
	Closure	-	-	-	-	-
Levelized material production costs* (USD/t)	LCE	13,934.5	13,833.8	13,645.6	13,929.6	13,578.0
	Co	32,156.5	31,924.2	31,489.8	32,144.5	31,333.8
	Ni	16,078.3	15,962.1	15,744.9	16,072.3	15,666.9
	Mn	2,143.8	2,128.3	2,099.3	2,143.0	2,088.9

Supplementary Table 17. Facility design characteristics, input/output data, total-lifetime costs, and levelized material production costs for the hydrometallurgical upcycling facility as modelled in this study. *Levelized material production costs are based on market value-based cost allocation

Hydrometallurgical Upcycling Facility						
Facility outlet/ process design	Output metal ratio	NMC 95	NMC 811	NMC 622	NMC 532	NMC 111
	Lifetime	20 years				
	Availability	8000 hr/year				
	Capacity	2,000 t black mass/year				
	Input black mass ratio	NMC 721	NMC 721	NMC 721	NMC 721	NMC 721
Facility lifetime output (t)	LiOH x H_2O	7,135.6	7,135.6	7,135.6	7,135.6	7,135.6
	pCAM	211,355.4	52,688.4	39,534.5	45,952.4	69,153.4
	Na_2SO_4	588,890.5	146,803.5	110,153.2	128,035.2	192,679.2
Facility lifetime costs (million USD)	CapEx/SusEx	139.0	139.0	139.0	139.0	139.0
	OpEx	2,347.3	512.0	362.1	416.4	786.5
	Closure	-	-	-	-	-
Levelized material production costs* (USD/t)	LCE	6,578.4	6,516.0	6,463.1	6,506.3	6,945.4
	pCAM	11,286.2	11,300.2	11,372.5	10,918.7	12,455.6

Supplementary Table 18. Consumption data and costs of the hydrometallurgical recycling plant.

OpEx catgory	Type	Price	Total-lifetime consumption for output according to target NMC metal ratio NMC 95	NMC 811	NMC 622	NMC 532	NMC 111
Reagent	Black mass	*(see Table 21)*	35,000.0 t/a	35,000.0 t/a	35,000.0 t/a	35,000.0 t/a	35,000.0 t/a
	Sulfuric Acid (H_2SO_4)	120.0 USD/t	82,556.8 t/a	82,556.8 t/a	82,556.8 t/a	82,556.8 t/a	82,556.8 t/a
	Hydrogen peroxide (H_2O_2)	340.0 USD/t	4,660.5 t/a	4,660.5 t/a	4,660.5 t/a	4,660.5 t/a	4,660.5 t/a
	Sodium hydroxide (NaOH)	345.0 USD/t	94,174.4 t/a	94,174.4 t/a	94,174.4 t/a	94,174.4 t/a	94,174.4 t/a
	Sodium carbonate (Na_2CO_3)	190.0 USD/t	17,918.8 t/a	17,918.8 t/a	17,918.8 t/a	17,918.8 t/a	17,918.8 t/a
	Quicklime (CaO)	124.0 USD/t	7,432.7 t/a	7,432.7 t/a	7,432.7 t/a	7,432.7 t/a	7,432.7 t/a
	Sodium hydrosulfide hydrate ($NaSH \times H_2O$)	300.0 USD/t	803.5 t/a	803.5 t/a	803.5 t/a	803.5 t/a	803.5 t/a
	D2EHPA	777.2 USD/t	24.5 t/a	24.5 t/a	24.5 t/a	24.5 t/a	24.5 t/a
	Cyanex272	31,800.0 USD/t	21.0 t/a	21.0 t/a	21.0 t/a	21.0 t/a	21.0 t/a
	PC88A	8,600.0 USD/t	17.5 t/a	17.5 t/a	17.5 t/a	17.5 t/a	17.5 t/a
Utilities	Electricity	0.133 USD/kWh	208,312 MWh/a	208,312 MWh/a	208,312 MWh/a	208,312 MWh/a	208,312 MWh/a
	Cooling Water	0.690 USD/m^3	6,959,098 m^3/a	6,959,098 m^3/a	6,959,098 m^3/a	6,959,098 m^3/a	6,959,098 m^3/a
	Steam	9.20 USD/t	104,253 t/a	104,253 t/a	104,253 t/a	104,253 t/a	104,253 t/a
	Wastewater	0.005 USD/gal	513,196,481 gal/a	513,196,481 gal/a	513,196,481 gal/a	513,196,481 gal/a	513,196,481 gal/a
Total reagent and utility costs (million USD)			5,000.8	5,002.7	4,962.3	4,699.0	5,008.5
Total other OpEx costs (million USD)			624.8	624.8	624.8	624.8	624.8
Total CapEx/SusEx (million USD)			1,347.6	1,347.6	1,347.6	1,347.6	1,347.6
Total closure costs (million USD)			-	-	-	-	-
**Total-lifetime costs (million USD)**			**6,973.2**	**6,975.1**	**6,934.8**	**6,671.4**	**6,980.9**

Supplementary Table 19. Consumption data and costs of the hydrometallurgical upcycling plant

OpEx catgory	Type	Price	Total-lifetime consumption for output according to target NMC metal ratio NMC 95	NMC 811	NMC 622	NMC 532	NMC 111
Reagent	Black mass	4,779 USD/t	2,000.0	2,000.0	2,000.0	2,000.0	2,000.0
	Sulfuric Acid (H2SO4)	120.0 USD/t	1,017.1	1,017.1	1,017.1	1,017.1	1,017.1
	Sulfur dioxide (SO2)	233.3 USD/t	862.6	862.6	862.6	862.6	862.6
	Sodium hydroxide (NaOH)	345.0 USD/t	21,009.6	5,237.4	3,929.9	4,567.9	6,874.1
	Ammonium hydroxide (NH_4OH)	1,200.0 USD/t	6,337.9	1,580.0	1,185.5	1,378.0	2,073.7
	Manganese sulfate ($MnSO_4 \times H_2O$)	700.0 USD/t	110.5	110.5	343.2	887.4	1,796.1
	Nickel sulfate ($NiSO_4 \times 6H_2O$)	3,500.0 USD/t	25,785.4	2,753.0	-	-	-
	Cobalt sulfate ($CoSO_4 \times 7H_2O$)	7,500.0 USD/t	-	-	387.1	637.9	2,803.3
	Water (H_2O)	15.0 USD/t	6,892.9	6,892.9	6,892.9	6,892.9	6,892.9
Utilities	Electricity	0.133 USD/kWh	59,398 kWh/a	59,398 kWh/a	59,398 kWh/a	59,398 kWh/a	59,398 kWh/a
	Cooling Water	0.690 USD/m^3	397,663 m^3/a	397,663 m^3/a	397,663 m^3/a	397,663 m^3/a	397,663 m^3/a
	Wastewater	0.005 USD/gal	26,390,800 gal/a	26,390,800 gal/a	26,390,800 gal/a	26,390,800 gal/a	26,390,800 gal/a
Total reagent and utility costs (million USD)			2,311.6	476.3	326.4	380.7	750.8
Total other OpEx costs (million USD)			35.7	35.7	35.7	35.7	35.7
Total CapEx/SusEx (million USD)			139.0	139.0	139.0	139.0	139.0
Total closure costs (million USD)			-	-	-	-	-
**Total-lifetime costs (million USD)**			**2,486.3**	**651.0**	**501.1**	**555.4**	**925.5**

Supplementary Table 20. Applied metal contents in various NMC black mass inputs based on Benchmark Mineral Intelligence data and molar mass-based recalculations. *NMC 721 is included as input material for hydrometallurgical upcycling facility.

Metal price scenario	Black mass metal contents (in wt%) NMC 95	NMC 811	NMC 622	NMC 532	NMC 111	NMC 721*
Li	5.0%	5.0%	5.0%	5.0%	5.0%	5.0%
Ni	34.6%	29.2%	22.0%	18.2%	12.1%	25.5%
Co	0.9%	3.7%	7.0%	7.3%	12.2%	5.5%
Mn	0.9%	3.4%	7.0%	10.2%	11.4%	5.1%

Supplementary Table 21. Calculated black mass prices (in USD/t) for various NMC ratios and metal price scenarios (baseline, moderate, minimum). *NMC 721 is included as input material for hydrometallurgical upcycling facility.

Metal price scenario	Black mass metal price (in USD/t) with metal content according to NMC chemistry					
	NMC 95	NMC 811	NMC 622	NMC 532	NMC 111	NMC 721*
Baseline	4,775	4,778	4,720	4,344	4,786	4,779
Moderate	3,235	3,292	3,320	3,076	3,468	3,329
Minimum	2,172	2,195	2,196	2,029	2,269	2,211

Supplementary Table 22. Calculated aggregated material cost for NMC CAM production (in USD/GWh CAM) supplied by hydrometallurgical recycling and upcycling facilities for baseline, moderate, and minimum metal price scenarios.

Recycling facility	Metal price scenario	Aggregated costs (USD/GWh CAM)				
		NMC 95	NMC 811	NMC 622	NMC 532	NMC 111
Hydrometallurgical recycling	Baseline	18,180,019	19,691,566	21,990,386	22,105,044	24,442,062
	Moderate	15,448,366	16,837,144	18,969,975	19,247,398	21,302,411
	Minimum	13,562,347	14,731,140	16,545,834	16,888,687	18,445,287
Hydrometallurgical upcycling	Baseline	15,568,214	16,824,810	18,761,009	18,480,725	22,245,183
	Moderate	11,975,903	13,902,946	15,915,685	15,562,152	17,681,031
	Minimum	8,466,632	11,323,815	13,862,755	13,519,434	14,835,183

Supplementary Table 23. Total end-of-life NMC battery supply in the United States for 2025 to 2050 in a low, basic, and high supply scenario as defined in this study.

Year	Scenario EoL NMC battery supply (in kWh)		
	Low	Basic	High
2025	295,657	295,657	295,657
2026	891,365	891,365	891,365
2027	2,003,129	2,003,136	2,003,143
2028	3,332,453	3,332,745	3,333,040
2029	4,955,729	4,960,185	4,964,689
2030	8,210,165	8,248,849	8,287,900
2031	14,750,678	14,983,622	15,218,464
2032	24,559,712	25,634,987	26,717,519
2033	34,337,049	38,104,204	41,891,157
2034	43,669,419	54,118,525	64,602,291
2035	56,173,558	77,874,636	99,581,227
2036	76,688,751	110,285,658	143,758,967
2037	102,846,365	147,912,299	192,765,054
2038	131,011,541	188,088,036	245,050,483
2039	158,605,823	227,344,041	296,163,186
2040	180,921,802	259,054,050	337,381,586
2041	194,640,661	278,545,386	362,600,574
2042	204,509,553	292,695,693	380,765,371
2043	215,273,927	308,128,312	400,635,078
2044	227,715,002	325,761,098	423,533,771
2045	242,191,581	346,162,840	450,078,839
2046	259,291,785	370,424,345	481,646,508
2047	279,009,046	398,462,086	518,248,368
2048	301,336,192	430,263,015	559,749,103
2049	327,608,814	467,635,922	608,323,831
2050	360,174,444	514,018,101	668,512,734

Supplementary Note 1. NMC cathode active material (CAM) production data for material cost aggregation

Throughout this study, we analyze and compare lithium, cobalt, and nickel supply configuration costs on the level of aggregated material costs that are required for the production of one GWh of NMC ($LiNi_xMn_yCo_zO_2$) cathode active material (CAM) in a 400-mile electric vehicle (EV). Parameter for battery characteristics, as well as bill-of-materials (i.e., material intensities) for the production of different NMC chemistries (see Supplementary Table 1) are sourced from Argonne National Laboratory' GREET model.[1,2]

In the state-of-the art production pathway of NMC CAMs, first, a precursor CAM (pCAM) material ($(Ni_xMn_yCo_z)(OH)_2$) is produced by mixing cobalt, nickel, and manganese metal sources ($CoSO_4$, $NiSO_4$, $MnSO_4$) according to target metal ratios of NMC CAM (e.g., NMC 95, 811, 622, 532, or 111), followed by a co-precipitation with sodium hydroxide (NaOH) and ammonium hydroxide (NH_4OH), and filtration, washing, and drying of produced pCAM. Second, pCAM is mixed with a lithium source (Li_2CO_3 or LiOH), followed by multi-stage calcination process at temperatures up to 1000°C for up to 12 hours, before final product is crushed and sieved. Finally, the NMC CAM undergoes a metal detection and demagnetization process, after which the produced battery-grade NMC CAM can be used in downstream cell and battery manufacturing stages.[1]

Supplementary Note 2. Primary company data: Global lithium, cobalt and nickel mining and refining facilities

To calculate aggregated material costs for the production of NMC CAM, instead of market prices, our model uses levelized material production costs (USD/t) for lithium (as Li_2CO_3 or LiOH), cobalt (as $CoSO_4$), nickel (as $NiSO_4$), and manganese (as $MnSO_4$). We draw on primary data of more than 80 company reports on mining, refining, and battery-grade material production (conversion) plants to derive levelized production costs for lithium, cobalt, and nickel. Supplementary Table 2 shows a conclusion of levelized material production costs for all lithium, cobalt, and nickel mining/refining facilities analyzed in this study, including by-product credits (see Supplementary Note 3) and all costs up to final battery-grade input materials for NMC CAM synthesis. A detailed listing of all mining and refining facilities, including ownership, location, deposit type, applied process, product type, by-products, lifetime, supply capacity, and cost structure, are presented in the Supplementary Sheet. Primary company data for obtaining levelized manganese production costs, as well as cost data for NaOH and NH_4OH are shown in Supplementary Note 4. Furthermore, this study's primary data set of conversion plants and complementary techno-economic modelling to derive conversion regional costs is discussed in Supplementary Note 5.

Supplementary Note 3. By-product cost allocation methodology: Market value-based cost allocation (including recycling)

Most of the cobalt and nickel mines/refineries, as well as some lithium mines/refineries and the recycling facility analyzed in this study (see Supplementary Note 7) produce by-products. In these cases, we determine levelized production costs for each by-product by a market value-based cost allocation[4] method, which allocates costs based on total revenue shares. More specifically, based on individual facility's total-lifetime supply capacity (in t) and average market price (USD/t) of each by-product (see Supplementary Table 3), we calculate total-lifetime revenue and by-product revenue shares of total revenue. Each by-product's total revenue share is then applied for cost allocation of total-lifetime facility cost to individual by-products. However, some company reports

specifically report on by-product credits. In these cases, we adapted final costs by reported by-product credits in primary company data.

Supplementary Note 4. Cost data for other CAM production input materials: Manganese, sodium hydroxide, and ammonium hydroxide

Because of marginal contribution to the bill-of-material of NMC production and aggregated cost, throughout the analyses of different supply sources and across all NMC chemistries, unit costs (USD/t) for NaOH and NH4OH are kept constant at 345.0 USD/t and 1,200.0 USD/t, respectively.[5,6] Furthermore, for our U.S. domestic and all foreign primary supply scenarios based on mining and refining facilities, unit costs for manganese (as $MnSO_4$) are help constant as well. However, similar to our analysis of levelized production costs for lithium, cobalt, and nickel, we analyzed primary company data for three manganese mines and refineries, which incorporate battery-grade $MnSO_4$ production. To derive global average levelized manganese production costs, we averaged identified facility-individual levelized costs by facilities' total-lifetime supply (see Supplementary Table 4), resulting in a global average of 1,694.02 USD/t Mn (as $MnSO_4$).

Supplementary Note 5. Conversion to battery-grade materials: Primary company data, complementary techno-economic modelling, and average regional conversion costs

To obtain costs for total production of battery-grade Li_2CO_3/LiOH, $CoSO_4$, and $NiSO_4$, we analyzed levelized conversion costs based on primary company data of four refineries, which are shown in Supplementary Table 5. Furthermore, we complemented the derived levelized conversion costs with further data from other reports and techno-economic modelling of low-cost refineries (e.g., located in China) based on our primary data. S&P Global Market Intelligence reports average lithium conversion costs from spodumene concentrate to LiOH monohydrate of 2,456.0 USD/t LCE for global conversion facilities[7] that are mainly based in China,[8] which we added as low-cost facilities to the data set. To further obtain low-cost cobalt and nickel conversion costs of global cobalt and nickel conversions, which are mainly located in China as well,[8] we modelled Chinese conversion costs based on our primary data on cobalt and nickel sulfate production costs. In doing so, we reduced total CapEx, SusEx and closure costs by 30%, and adapted OpEx according to lower reagent, utility, and labor costs in China compared to our primary data based on plants located in Canada. Reagent costs for Chinese conversion plants are modelled to amount to 40.8 USD/t sulfuric acid,[9] 216.0 USD/t NaOH,[5] and 350.0 USD/t calcium carbonate[10], which are the main driver of reagent costs in conversion processes. Utility costs are around 0.109 USD/kWh in primary company reports for facilities in Canada, while Chinese average utility costs are set at 0.087 USD/kWh.[11] Finally, labor costs are proportionally down-scaled according to average Canadian wages in the manufacturing sector[12] and average Chinese wages in the manufacturing sector.[13] Results for Chinese-based levelized conversion costs according to techno-economic re-modelling as described above are 5,844.2 USD/t Co and 1,549.9 USD/t Ni.

To ensure levelized production costs up to battery-grade Li_2CO_3/LiOH, $CoSO_4$, and $NiSO_4$ we added these costs to the levelized production costs of mines/refineries that do not produce battery-grade materials, but intermediate products, such as spodumene concentrate, cobalt hydroxide, nickel concentrate etc. Furthermore, we assume regional conversion costs according to Supplementary Table 6. In this study, we assume low-cost conversion in to low-cost regions (Oceania, South America, Africa), reflecting current industrial practices of dominated export of intermediate products to Chinese conversion facilities, and high-cost conversion in high-cost

regions (Canada, USA, Europe), reflecting current low-share refining in these regions and an increasing installation of domestic conversion capacity. Furthermore, we apply additional conversion costs to all mine/refinery levelized production costs that produce intermediate products and assume constant conversion costs across all intermediate products.

Supplementary Note 6. Average regional levelized production costs and aggregated costs for U.S. and Europe supply scenarios.

In this study, we allocate facility costs to supply regions to investigate aggregated CAM material costs based on different regional lithium, cobalt, and nickel supply scenarios. Regions as defined in this study including number of facilities and average regional supply costs are shown in Supplementary Table 7. Here, average regional material supply costs are determined as the LOM capacity-weighted average of all levelized material production in this region. Furthermore, Supplementary Table 8 and 9 show aggregate costs of an all-U.S. and Europe-based supply scenario as two regional supply scenario that we benchmark against the global average supply scenario (see Supplementary Note 7). For the aggregated costs of the European supply scenario, due to missing cost data on European nickel supply, we assume an average levelized nickel production cost-level of the average Canadian nickel supply, whose facilities show similar deposit types and process economics (e.g., CapEx intensity, labor costs etc.).

Supplementary Note 7. Definition of average global supply scenario costs: Global battery material supply data

The average global supply scenario is defined according to 2024 country supply chares of total global lithium, cobalt, and nickel supply (see Supplementary Table 10-12), excluding the United States. For further supply information, we also included data on manganese supply (Supplementary Table 13), although manganese production costs are held constant across all scenarios and NMC chemistries (see Supplementary Note 4). Due to missing data for levelized material production costs of individual countries (e.g., China, Russia, etc.), all these countries are added 'other' country supply in our global supply scenario definition (see Supplementary Table 14). However, levelized material production costs for 'other' countries is determined by the LOM capacity-weighted levelized material production costs of all remaining mines/refineries in our data set. For lithium, for example, 'other' countries production costs are determined by the LOM capacity-weighted average costs of mines/refineries in Mali, Ghana, Germany, Czech, Austria, Finland, Spain, Namibia, DR Congo, Mexico, and the United States. Resulting aggregated material costs for NMC CAM production in the global supply scenario is shown in Supplementary Table 15.

Supplementary Note 8. Recycling costs: Primary company data and techno-economic modelling

Levelized production costs for materials (Li_2CO_3/LiOH, $CoSO_4$, $NiSO_4$, $MnSO_4$, and pCAM) sourced from recycling is derived from primary company data and complemented by techno-economic modelling. Here, we draw on primary data of two recycling facilities: a hydrometallurgical recycling and a hydrometallurgical upcycling facility. First, levelized material production costs from hydrometallurgical recycling is modelled based on primary data on the company Li-Cycle's hydrometallurgical refining plant in Rochester, New York, which is designed to convert black mass into Li_2CO_3, $CoSO_4$, $NiSO_4$, and $MnSO_4$. Second, levelized material production costs from hydrometallurgical upcycling is modelled based on primary data on a plant by the company RecycLiCo, which offers a modular manufacturing and on-site installation of

recycling plants to customer. For both facilities, we identified primary data for initial CapEx (equipment costs/total cost-of-installation), input/reagent consumption, and utility consumption, which represent the majority of total-lifetime facility costs. In addition, we complemented missing costs data by techno-economic modelling based on Argonne National Laboratory's EverBatt model[14] and prior literature on recycling facility costs in the United States.[15] For both facilities, we apply a total lifetime of 20 years based on literature,[15] and assume no occurring closure costs of the facilities. Details on primary cost data and techno-economic modelling for both recycling facilities is described in the following.

Hydrometallurgical recycling facility: Canada-based Li-Cycle's hydrometallurgical recycling plant in Rochester, New York (so-called 'Rochester Hub') is designed to be capable of processing 35,000 tons of black mass per year into battery-grade around 8,500 tons of Li_2CO_3, 7,500 tons of $CoSO_4$ (heptahydrate), and 48,000 tons of $NiSO_4$ (hexahydrate) per year and corresponding amounts of a manganese salts,[16,17] which amount to around 4,500 tons per year $MnSO_4$ monohydrate equivalents. Most recent company strategies propose a production of around 8,250 tons of Li_2CO_3 per year and a mixed-hydroxide precipitate (MHP) with a rate of 72,000 tons per year, containing cobalt, nickel, and manganese. However, in this study, our modelling is based on former sulfate salt production data, which is corresponding to current company data on inherent cobalt (ca. 1,600 tons per year) and nickel (ca. 11,300 tons per year) in the MHP.[17] Initial capital expenditures (CapEx) for total cost-to-installation of the recycling facility accounts for 960.2 million USD, according to Li-Cycle's most recent company reports.[18] We assume these costs as total direct costs as input into the EverBatt model. Total-lifetime CapEx (including SusEx) is then calculated by the EverBatt model, resulting in 1.347.6 million USD of total capital investment (as defined in EverBatt). We group recycling operating expenditures (OpEx) into black mass costs, reagent costs, utility costs, and other OpEx (including fixed OpEx, such as labor etc.), which are shown in Supplementary Table 18. Reagent and utility consumption data are obtained from public data provided by the U.S. Department of Energy's Loan Programs Office.[19,20] Missing data for additional reagents (e.g., solvent extraction agents), utility consumption (e.g., waste water), and other OpEx is sourced from literature values for a U.S. hydrometallurgical recycling plant with a capacity 8,000 tons of black mass per year,[15] and proportionally scaled towards the processing capacity of Li-Cycle's facility. Consumption data and total-lifetime costs of the hydrometallurgical recycling facility is shown in Supplementary Table 18. As a base case, Li-Cycle's reports on facility output data imply an input black mass and output metal ratio according to NMC 811. In recalculation towards other NMC chemistries, we apply molar masses to input black mass contents and output metal sulfate salts (input black mass equals output metal ratios of cobalt, nickel, and manganese), while keeping Li_2CO_3 output rates and consumption rates of reagents constant. All input and output data of the hydrometallurgical recycling facility processing black mass and producing metal salts according to all five NMC chemistries is shown in Supplementary Table 16.

Hydrometallurgical upcycling facility: The modelling of the hydrometallurgical upcycling facility by Canada-based company RecycLiCo is based on primary company reports on a facility installed by RecycLiCo with a nameplate capacity of 2,000 tons of black per year.[21] RecycLiCo applies a hydrometallurgical upcycling process that converts input black mass to LiOH and pCAM output.[22] By adding dopant metal sulfates (e.g., $CoSO_4$ or $MnSO_4$), the process is flexibly adaptable towards target output pCAM NMC chemistries.[6] Our modelling is based on primary data for initial CapEx for facility equipment and reagent and electricity consumption rates, complemented by techno-economic modelling of other utility consumption rates, other OpEx, and

total CapEx/SusEx by the EverBatt model and literature. Equipment costs of RecycLiCo's model facility amount to around 35 million USD,[21] which result in 139 million USD of total capital investment costs with modelling by EverBatt. Primary reagent and electricity consumption data are gathered for a base case black mass input with a metal ratio of approx. NMC 721 and an output pCAM according to a metal ratio of NMC 532.[6] In this study, for other target pCAM NMC chemistries, we assume a constant reported black mass input (approx. NMC 721 metal ratio; 2,000 tons per year processed black mass) and conduct a molar mass-based recalculation of required amount of dopant agents ($CoSO_4$, $NiSO_4$, $MnSO_4$) and affected reagents (NaOH, NH_4OH), as well as output pCAM rates towards the remaining four NMC chemistries analyzed in this study (NMC 95, 811, 622, and 111). However, LiOH production rates remain unaffected during recalculation because black mass input is constant across NMC chemistries and no further lithium is added during the doping processes in the facility. Equal to the complementary techno-economic modelling of the hydrometallurgical recycling facility by Li-Cycle, missing data on other utilities (e.g., steam or waste water) and other OpEx (e.g., labor) are complemented by proportional down-scaling of data obtained from literature (U.S. hydrometallurgical recycling plant for processing 8,000 tons black mass per year).[15] A conclusion of hydrometallurgical upcycling consumption data and total-lifetime costs is shown in Supplementary Table 19. Furthermore, key facility input and output data of the hydrometallurgical upcycling facility producing all five NMC chemistries is shown in Supplementary Table 17.

Supplementary Note 9. Black mass price modelling

In this study, we model black mass prices for different NMC based on inherent metal value of lithium, cobalt, and nickel, while manganese does not contribute to black mass value (0% payable). Overall, we model the black mass price (BMP; in USD/t) in the United States as a function of black mass metal contents (M; in t), metal payables (P; in %), and metal market prices (Y; in USD/t), according to the following supplemental equation 1:

$$BMP_{n,t}\left[\frac{USD}{t}\right] = \sum_{i}\left(M_i^n\,[\mathrm{t}] * P_{i,t}\,[\%] * Y_{i,t}\left[\frac{USD}{t}\right]\right) \tag{1}$$

where i is the metal (lithium, cobalt, nickel, or manganese), n is the NMC chemistry of the black mass, and t is the respective year. We draw on data for recent black mass price and metal payable developments in North America provided by Benchmark Mineral Intelligence.[23] Baseline case black mass prices are calculated based on material market value according to Supplementary Table 3 and average payables of 80% for all metals. Metal contents are based on molar mass-based recalculation of data from Benchmark Mineral Intelligence,[23] and concluded in Supplementary Table 20.

In the black mass price scenario analysis of this study, we adapt the black mass prices for each NMC ratio resulting from lowered metal price scenarios. In the moderate metal price scenario, metal costs are lowered to 7,500 USD/t lithium, 22,500 USD/t cobalt, and 10,000 USD/t nickel. In the minimum metal price scenario, metal prices approach minimum price thresholds defined as the minimum average regional material production costs derived from the primary data set on mines/refineries. For lithium, minimum prices in this study are 4,632.3 USD/t based on Australia's average production costs. For cobalt and nickel, the minimum prices are 14,588.3 USD/t (DR Congo) and 6,784.9 USD/t (Papua New Guinea), respectively.

Correspondingly, in the moderate and minimum black mass prices, metal sulfate salts prices for the hydrometallurgical upcycling facility are lowered. Baseline case metal sulfate salt prices are 7,500 USD/t $CoSO_4$ and 3,500 USD/t $NiSO_4$. Our moderate price scenario metal sulfate salt prices are set at 4,750 USD/t $CoSO_4$ and 2,500 USD/t $NiSO_4$, and minimum metal sulfate prices at 3,500 USD/t $CoSO_4$ and 1,500 USD/t $NiSO_4$. Supplementary Table 21 shows an overview of black mass prices across all scenarios and NMC chemistries. Resulting aggregated costs of all five NMC chemistries for materials sourced from the hydrometallurgical recycling and upcycling plants are shown in Table Supplementary 22.

Supplementary Note 10. Learning effects or economies-of-scale: Wright's Law

Due to limited availability of recyclable, end-of-life (EoL) battery supply, recycling processes and facilities are still at an early stage of technology maturation processes. Thus, cost optimizations of the hydrometallurgical recycling and upcycling facilities' CapEx and process costs caused by economies-of-scale are expected to occur with growing battery deployment in electric vehicles, EoL battery supply, and demand for recycled materials. In prior academic literature, quantifications of such learning effects (or economies-of-scale) are commonly based on Wright's Law, which determines cost reduction potentials by in-time learning effects of immature technologies with increasing market adoption and production capacity expansion.[24] In this study, we apply Wright's Law based on the two recycling facilities' cost structures. We group total costs for the hydrometallurgical recycling plant into black mass input costs and remaining costs (including CapEx, SusEx, other reagents, utilities, labor, and other OpEx), whereas for the hydrometallurgical upcycling plant, we group total costs into costs for black mass and metal sulfate salt inputs and remaining costs. In the expression of Wright's Law in this study, we apply a black mass and metal sulfate salt cost floor and a 10% reduction rate (i.e., learning rate) of remaining costs each time the recycling capacity is doubled.

Our model assumes the projected installed hydrometallurgical recycling and upcycling capacity in the United States to increase proportionally with the growing black mass supply in the United States, which, in turn, is driven by the U.S. EoL NMC battery supply. Thus, we apply a forecast model to derive future EoL NMC battery supply in the United States, by which installed recycling capacity is scaled (see Supplementary Note 11). The following supplemental equation 2 expresses Wright's Law as applied in this study:

$$Costs_t^{total} = \left(Costs_0^{total} - Costs_{floor}\right) * \left(\frac{EoL_t^{NMC}}{EoL_0^{NMC}}\right)^{log_2(1-r)} + Costs_{floor} \quad (2)$$

where $Costs_t^{total}$ are the total-lifetime recycling facility's costs at the time t, $Costs_0^{total}$ are the baseline total-lifetime recycling facility's costs, EoL_t^{NMC} is the total EoL NMC battery supply in the United States at the time t, EoL_0^{NMC} is the total baseline EoL NMC battery supply in the United States at the time $t = 0$ (year 2025 in this study), $Costs_{floor}$ is the cost floor consisting of black mass and metal sulfate salt costs, and r is the learning rate.

Supplementary Note 11. U.S. end-of-life (EoL) battery supply modelling

The forecast model to determine EoL battery supply for NMC batteries in the United States as applied in this study is based on prior research.[25,26] Within this model, we update parameter values according to historic U.S. full-electric vehicle and plug-in hybrid electric vehicle sales,[27] while adopting projected U.S. vehicle sales numbers and full-electric vehicle/plug-in hybrid electric

vehicle shares from the reference model. Furthermore, we assumed increased average battery sizes ranging from 66 kWh in 2017 (start year of the model) to 115.5 kWh for full-electric vehicles, while batteries for plug-in hybrid electric vehicles remain constant at 16 kWh over time. Finally, modelled collection rates for NMC batteries (aggregated NMC 111, NMC 532, NMC 622, NMC 622 (Graphite/Si-anode), NMC 811 (Graphite/Si-anode), and NMC 955-(Graphite/Si-anode) as defined in battery technology roadmap in reference model[25,26]) are assumed to increase from 50% in 2017 to 98% in 2050. Battery characteristics, such as energy density and NMC CAM intensity are derived from the GREET model. In this study, we apply a basic scenario derived from parameter values as described above, and a low and high U.S. EoL NMC battery supply scenario, in which projected full-electric vehicle sales are decreased/increased by 30%, respectively. Total EoL NMC battery supply for all three scenarios (in kWh) is presented in Supplementary Table 21.

Supplementary References

1 Iyer, R. & Kelly, J. Updates to lithium-ion battery material composition for vehicles. *Argonne National Laboratory, Lemont, IL* (2023).
2 Dai, Q., Kelly, J. C., Dunn, J. & Benavides, P. T. Update of bill-of-materials and cathode materials production for lithium-ion batteries in the GREET model. *Argonne National Laboratory, Lemont, IL* (2018).
3 USGS. Mineral Commodity Summaries, https://www.usgs.gov/centers/national-minerals-information-center/mineral-commodity-summaries, (2025).
4 Valero, A., Domínguez, A. & Valero, A. Exergy cost allocation of by-products in the mining and metallurgical industry. *Resour. Conserv. Recycl.* **102**, 128-142 (2015).
5 Sodium hydroxide price index, https://businessanalytiq.com/procurementanalytics/index/sodium-hydroxide-price-index/, (2025).
6 Jung, J., Sui, P.-C. & Zhang, J. *Hydrometallurgical recycling of lithium-Ion battery materials*. (CRC Press, 2023).
7 Lithium sector: production costs outlook, https://pages.marketintelligence.spglobal.com/lithium-sector-outlook-costs-and-margins-confirmation-CD.html, (2019).
8 Cheng, A. L., Fuchs, E. R. H., Karplus, V. J. & Michalek, J. J. Electric vehicle battery chemistry affects supply chain disruption vulnerabilities. *Nat. Commun.* **15**, 2143 (2024).
9 Intratec. Sulfuric Acid Prices Worldwide, https://www.intratec.us/solutions/primary-commodity-prices/commodity/sulfuric-acid-prices, (2025).
10 IQ, B. A. Calcium carbonate price index, https://businessanalytiq.com/procurementanalytics/index/calcium-carbonate-price-index/, (2025).
11 Statista. Average electricity prices for enterprises in China from September 2019 to June 2024, https://www.statista.com/statistics/1373596/business-electricity-price-china/, (2025).
12 Canada, S. Employee wages by industry, annual. (2025).
13 Economics, T. China Average Yearly Wages in Manufacturing. (2025).
14 Dai, Q. *et al.* EverBatt: A closed-loop battery recycling cost and environmental impacts model. (Argonne National Lab.(ANL), Argonne, IL (United States), 2019).
15 Vu, T. T., Seo, J. & Song, D. A comprehensive techno-economic analysis of the full project for recycling valuable metals from waste Lithium-Ion battery. *J. Environ. Chem. Eng.* **12**, 114751 (2024).

16 Li-Cycle. Li-Cycle to Upsize Capacity of First Hub Facility in North America to Meet Accelerating Demand for Battery Recycling, https://investors.li-cycle.com/news/news-details/2021/Li-Cycle-to-Upsize-Capacity-of-First-Hub-Facility-in-North-America-to-Meet-Accelerating-Demand-for-Battery-Recycling/default.aspx, (2021).

17 Li-Cycle. Li-Cycle – Investor's relations: Investor Presentation: Year-End 2023 Earnings & Business Update Call, https://investors.li-cycle.com/overview/default.aspx, (2023).

18 Li-Cycle. Li-Cycle – 10-K Annual report for the fiscal year ended December 31, 2024 https://investors.li-cycle.com/financials/sec-filings/default.aspx, (2024).

19 DOE. Li-Cycle SEQRA Attachment F. Utility Service Supporting Doc https://www.energy.gov/lpo/li-cycle-seqra-supplemental-documents, (2021).

20 DOE. Li-Cycle SEQRA Attachment M. Chemical Volume and Storage https://www.energy.gov/lpo/li-cycle-seqra-supplemental-documents, (2021).

21 Recyclico. Investors Presentation, https://recyclico.com/investors/, (2024).

22 Recyclico. Technology, https://recyclico.com/technology/, (2024).

23 Black Mass Price Assessment, https://www.benchmarkminerals.com/recycling/black-mass-prices, (2025).

24 Yao, A., Benson, S. M. & Chueh, W. C. Critically assessing sodium-ion technology roadmaps and scenarios for techno-economic competitiveness against lithium-ion batteries. *Nat. Energy* **10**, 404-416 (2025).

25 Wesselkämper, J., Dahrendorf, L., Mauler, L., Lux, S. & von Delft, S. A battery value chain independent of primary raw materials: Towards circularity in China, Europe and the US. *Resour. Conserv. Recycl.* **201**, 107218 (2024).

26 Wesselkämper, J., Hendrickson, T. P., Lux, S. & von Delft, S. Recycling or Second Use? Supply Potentials and Climate Effects of End-of-Life Electric Vehicle Batteries. *Environ. Sci. Technol.* **59**, 15751-15765 (2025).

27 Department-of-Transportation. Hybrid-Electric, Plug-in Hybrid-Electric and Electric Vehicle Sales, https://www.bts.gov/content/gasoline-hybrid-and-electric-vehicle-sales, (2025).
