# Supplementary material for: Primary material supply configurations and domestic recycling for cost-effective battery material production in the US
Source: Nat Commun. 2025 Dec 9;17:253. doi: 10.1038/s41467-025-66957-5 (PMC12783756; doi:10.1038/s41467-025-66957-5)
Supplement: Supplementary file 2 — Description of Additional Supplementary File [file 41467_2025_66957_MOESM2_ESM.pdf]

## Description of Additional Supplementary File

**Supplementary Data 1-** References of all facilities used in this study, as well as XY values of all data use in the study for each figure and every panel.
